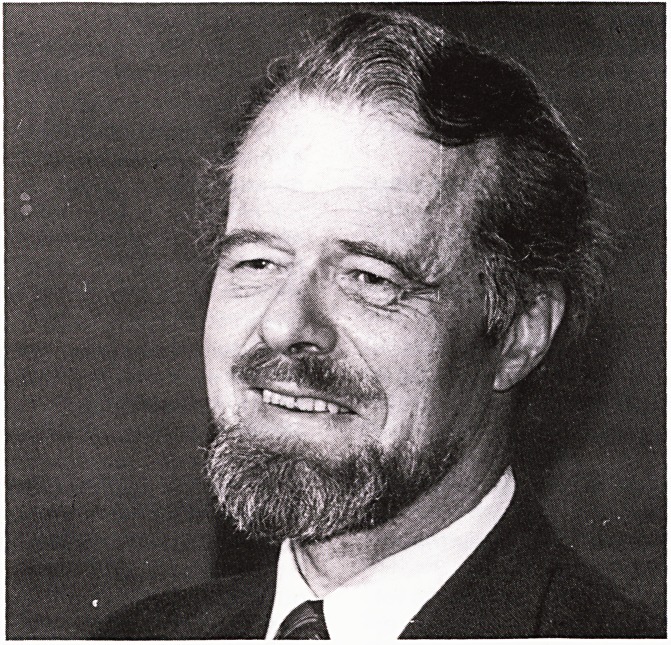# Dr Michael Lennard

**Published:** 1986-08

**Authors:** D. S. Ratcliffe


### Obituary

### Dr M. B. Lennard, MB, ChB (Bristol), FRCGP

Michael Briart Lennard, a member of the Editorial Committee of this Journal and who, with his wife Joan, was a loyal supporter of Bristol Medico-Chirurgical Society, died on 31 May aged 63.

Like others who set their hands to cultivate new ground—in his case the field of general practice—he was opposed by those who preferred wild pastures where they pointed to the rarer natural flowers but ingnored the vast areas of neglect. More than was generally realised, he was well aware of the antagonisms aroused by the College whose aims he did so much to nurture in its early days. But out of this College, with all its failings, came the gradual acceptance of a new discipline in family medicine. In Bristol, this acceptance was initially a cautious one, not only by general practitioners but by many consultants and by the University. As a consequence, a compromise 'Medicine-in-the-Community' course was started for students in 1971 in which Michael played a leading part.

Properly-organised vocational training was also started in the South West and in 1972 he was appointed Regional Adviser—a post which he filled with energy and distinction for ten years. He summarised the philosophy and evolution of the programme in a paper published in this Journal in July last year (Volume 100 (iii) Number 375 pp 72–3).

Both he and Joan taught in the practice they pioneered in Hartcliffe and Bishopswoth, and Michael was for many years an examiner for the RCGP—which experience must have given him valuable insight into the strengths and weaknesses of the new training. Both of them realised that, unless general practitioners themselves demonstrated new qualities as generalists, they could not expect to be taken seriously by specialists. Trainees readily caught Michael's enthusiasm and were to find this constantly reinforced by practical encouragement. From his example they recognised the importance of courtesy towards both patients and colleagues. His seminars were often enlivened by humour and his gift of parody.

He enjoyed painting and drawing and had always been a great reader—Anthony Trollope and Rudyard Kipling were among his favourite authors—and no doubt his love of art and literature contributed to the stoicism with which he bore his final illnesses but it was Joan who was his mainstay. It is to her and their family that our thoughts now turn.

D. S. Ratcliffe

## Figures and Tables

**Figure f1:**